# Metabolomic Effects of the Dietary Inclusion of *Hermetia illucens* Larva Meal in Tilapia

**DOI:** 10.3390/metabo12040286

**Published:** 2022-03-24

**Authors:** Bo Ye, Jian Li, Lijun Xu, Hui Liu, Manjun Yang

**Affiliations:** 1 College of Resources and Environment, Zhongkai University of Agriculture and Engineering, Guangzhou 510225, China; yebo@zhku.edu.cn (B.Y.); liuhui@zhku.edu.cn (H.L.); 2 Innovative Institute of Animal Healthy Breeding, Zhongkai University of Agriculture and Engineering, Guangzhou 510225, China; 3 China Institute of Veterinary Drug Control, Beijing 100081, China; lijian@ivdc.org.cn; 4 Tibet University of Tibetan Medicine, Lhasa 850000, China; xukaixk11@163.com; 5 Tibetan Key Laboratory of Veterinary Drug, Tibet Vocational Technical College, Lhasa 850030, China

**Keywords:** *Hermetia illucens* larvae meal (HLM), feed, *Oreochromis niloticus*, metabolome, metabolomic effect

## Abstract

Black soldier fly (*Hermetia illucens*) larvae meal have been used as feed protein supplements in fish feed, but few researches have investigated the metabolomic effects of *Hermetia illucens* larvae meal supplements. Therefore, the metabolic effects on Nile tilapia were investigated by replacing 5%, 10%, and 20% of the dietary soybean meal in the basal diet with *Hermetia illucens* larvae meal, respectively. This study shows that 20% *H. illucens* larvae meal feed could promote tilapia average daily gain of upto 5.03 ± 0.18 g (mean ± SEM). It was found that the tricarboxylic acid cycle efficiency was improved by activating the enzymes of mitochondrial isocitrate dehydrogenase, NAD-malate dehydrogenase, succinate dehydrogenase, pyruvate dehydrogenase, and α-ketoglutarate dehydrogenase, which then increased the output of ATP and NADH. Furthermore, amino acid and protein biosynthesis was boosted by enhanced glutamine synthetase and glutamate synthase. In particular, GSH increased with increased *H. illucens* larvae meal. Unsaturated fatty acid biosynthesis was stimulated by higher levels of fatty acid synthase and acetyl CoA carboxylase. Additionally, there was no significant change in lipase levels. Thus, the higher acetyl Co-A content was primarily involved in fatty acid biosynthesis and energy metabolism. Flavor substances, such as nonanal and 2-methyl-3-furanthiol, also accumulated with the addition of *H. illucens* larvae meal, which increased the umami taste and meat flavor. Additionally, the flavor of tilapia was improved owing to a decrease in trimethylamine content, which causes an earthy and fishy taste. This study uncovers a previously unknown metabolic effect of dietary *H. illucens* larvae meal on Nile tilapia.

## 1. Introduction

According to Food and Agriculture Organization of the United Nations, the most recent available estimates place global genetically-improved farmed tilapia production at approximately seven million tons by the end of 2020. The import and export value of tilapia is estimated to be approximately 615 million USD by 2021 (https://www.fao.org/in-action/globefish/market-reports/resource-detail/en/c/1416636/. (accessed on 29 January 2022)). Tilapias are cultured in more than one hundred tropical and subtropical countries, thereby improving fishing productivity and facilitating the development of aquaculture because of their high protein content, excellent growth performance, and adaptability to a wide range of rearing conditions [1,2,3,4]. Thus, Nile tilapia (*Oreochromis niloticus*) cultures are valuable for aquaculture worldwide. The shortage of feed protein resources is the major challenge to the world animal farming, and additionally the cost of feed protein resources is rising [5,6,7]. Therefore, there is an urgent need to find alternatives to feed protein resources. Perhaps, insect resources may be an effective response to the crisis [8,9,10,11,12,13,14,15,16,17].

*Hermetia illucens*, also known as the black soldier fly, is a true fly (*Diptera*) belonging to the *Stratiomyidae* family. Their adults consume nothing but water, do not approach humans, do not bite or sting, and do not vector or disseminate any specific diseases [8,9]. Their larval development time of over 21 days is longer than other flies such as house flies and carrion flies (less than 5 days), which means their larvae can consume more substrate [9]. Furthermore, their larvae can be fed with waste, such as manure [10], food waste [11], and kitchen waste [12]. The diversity of substrates they can process and the efficiency with which they do so exceeds that of most flies [13]. In addition, their larvae are rich in amino acids, proteins, fatty acids, minerals, and other nutrients [14]. Previously, studies have shown these larvae make excellent animal feed [15,16]. With these benefits, *H. illucens* larvae are a practical tool for efficient waste disposal as well as a sustainable animal feed, such as carp (*Cyprinus carpio*) [17] and tilapia (*O. niloticus*) [18].

*H. illucens* larva meal (HLM) is increasingly becoming a substitution for fish meal, and is increasingly becoming an important feed for carnivorous fish owing to its medium proteins and high fatty acids content [19]. The importance of fish meal and fish oil in aquaculture is well known, however factors such as competition with the demands for fish for human consumption and depleted fishery resources have lowered the supply of fish meal and fish oil and increased their cost, resulting in leading fisheries searching for alternatives such as insect meal and oils [20]. Studies have shown that larval feed cannot meet turbot (*Psetta maxima*) needs [19]; moreover, the addition of 25% larva meal to African catfish (*Clarias gariepinus*) feed did not significantly improve its production performance [21]. However, *H. illucens* larvae can make use of waste, therefore it is recommended as a feasible, partial supplements option for fish diets due to its high-quality nutrients. Finally, the ability of *H. illucens* larvae to efficiently produce protein-rich edible biomass from potentially protein-poor organic waste has led researchers to conclude that *H. illucens* larvae can contribute meaningfully to sustainable tilapia cultures as a partial or total meal replacement [22,23,24]. Unfortunately, few researches have been conducted to measure the metabolomic level of tilapias fed with HLM diet.

Recently, we developed a functional metabolomic approach for identifying crucial biomarkers and metabolic pathways [25,26]. Thus, the metabolic effects were investigated by replacing 5%, 10%, and 20% soybean meal in the basal diet with *H. illucens* larvae meal, respectively. Using the metabolomic approaches combined with enzymatic detection, these crucial biomarkers and pathways were used to determine the metabolomic effects of HLM diets on Nile tilapia, thereby improving feed efficiency and elevating the production performance of tilapia.

## 2. Results

### 2.1. H. illucens Larva Meal Boosting the Production Performance and Sensory Taste of Tilapia

**Figure 1:**
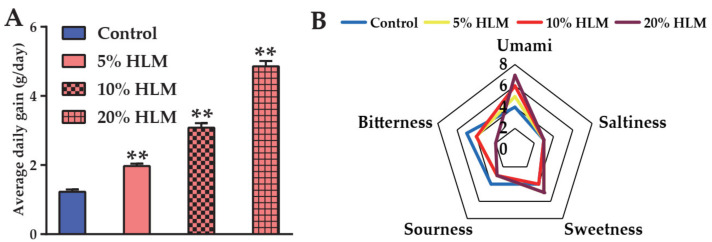
The average daily gain and sensory taste of Nile tilapia. (**A**) The average daily gain of tilapia fed with 5%, 10%, and 20% HLM feed compared with the basal diets. ** *p* < 0.01. (**B**) Shows the sensory taste of fish meat.

After feeding for 30 days, the average daily gain of tilapia fed with 5%, 10%, and 20% HLM diets were 1.97 ± 0.1, 3.21 ± 0.12, and 5.03 ± 0.18 g/day, respectively, compared to 1.23 ± 0.04 g/day (mean ± SEM) fed with basal diet (Figure 1A). Here, initial and final weight details were shown in Table S2. The results indicated that tilapia fed HLM were better than those in the basal diet control group. Furthermore, the meat sensory evaluation of these tilapias fed by basal diet, 5%, 10%, and 20% HLM feeds were shown in Figure 1B. It can be seen that control groups were mainly tasted as bitter and sour. The groups of 5%, 10%, and 20% HLM was dominated by umami and sweetness. With HLM increasing, the umami of tilapia meat was stronger than that fed basal diets, and the sweetness was also improved slightly. This result is consistent with the variation of the flavor metabolites, in which the flavor amino acids increased after HLM supplemented. It was concluded that HLM feed contribute to endow tilapia meat with better taste.

### 2.2. Metabolic Profile of Tilapias to H. illucens Larva Meal

**Figure 2:**
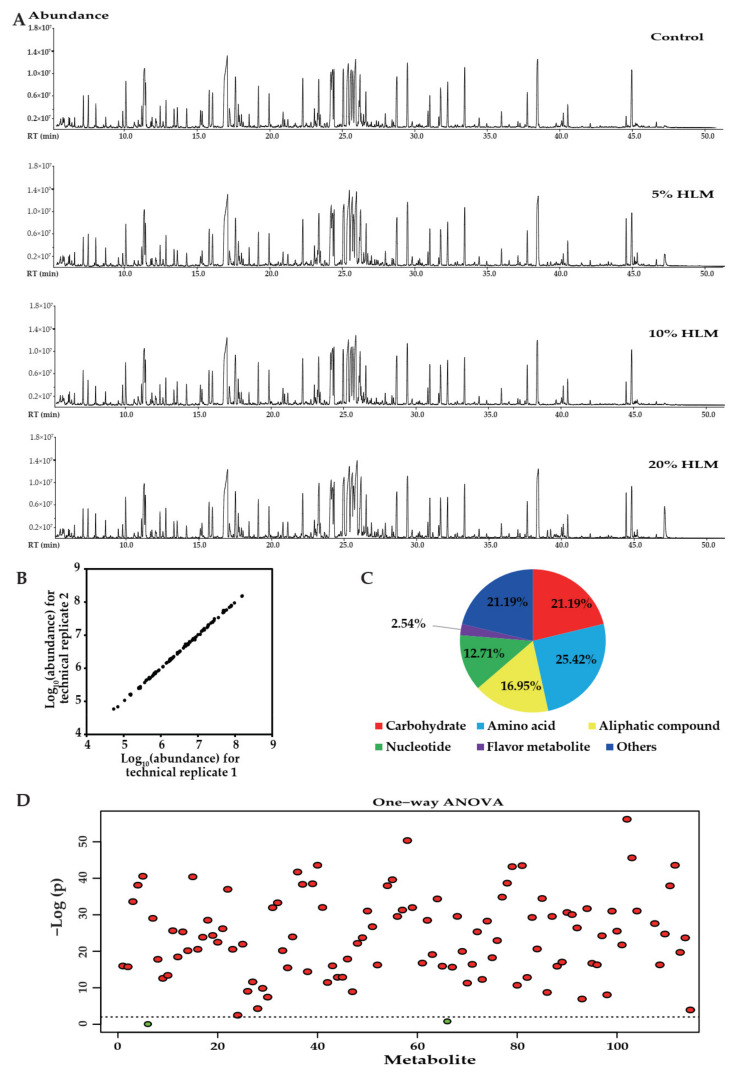
The different metabolism of tilapia. (**A**) Representative TIC from basal diet and HLM feed groups. (**B**) Reproducibility of metabolomic detection platform. Correlation of metabolites quantified in these groups over two technical replicates was shown. (**C**) Categories of the 118 metabolites that were searched in KEGG and NCBI PubChem Compound for their categories, and the pie chart was generated in Office Excel 2010. (**D**) One-way ANOVA significant map of different metabolites (row). Blue and red indicated non-significant and significant metabolites, respectively (*p* < 0.01).

The effects of HLM on the metabolome of tilapias were investigated as outlined in Figure 2. Livers were obtained from fish fed on the basal diet and 5%, 10%, and 20% HLM diets for 30 days and prepared for GC-MS-based metabolomics. Six biological individuals of four groups and two technical replicas from each group yielded 48 data. Representative total ion current chromatograms (TIC) from these groups were listed in Figure 2A, and 311 aligned individual peaks were obtained from each sample. The Pearson correlation coefficient between technical replicates varied between 0.9992 and 0.9999, demonstrating the reproducibility of the data (Figure 2B). According to annotation of KEGG (http://www.kegg.jp/. (accessed on 29 January 2022)) and NCBI PubChem (https://pubchem.ncbi.nlm.nih.gov/. (accessed on 29 January 2022)), the metabolites were classified into six categories, such as carbohydrates, amino acids, aliphatic compounds, nucleotides, and flavor substances. After mass spectra processing, a total of 118 metabolites were identified. Of these, the categorized proportion of various metabolites was 21.19% carbohydrates, 25.42% amino acids, 16.95% aliphatic compounds, 12.71% nucleotides, 2.54% flavor metabolites, and 21.19% others, respectively (Figure 2C). It was hypothesized that the differences in the metabolomes were related to the characteristic metabolomes constructed by these groups. To explore the metabolic biomarkers that distinguish these groups, one-way ANOVA coupled with a permutation test was used to identify the differential abundance of metabolites in the basal diet and 5%, 10%, and 20% HLM feed. There were 110 significant metabolites (*p* < 0.01) in these groups, which corresponded to a false discovery rate (FDR) of less than 0.003387 (Figure 2D). This is the FDR of citric acid and the largest FDR among the 110 metabolites.

### 2.3. Differential Metabolomes Responsible for H. illucens Larva Meal

**Figure 3:**
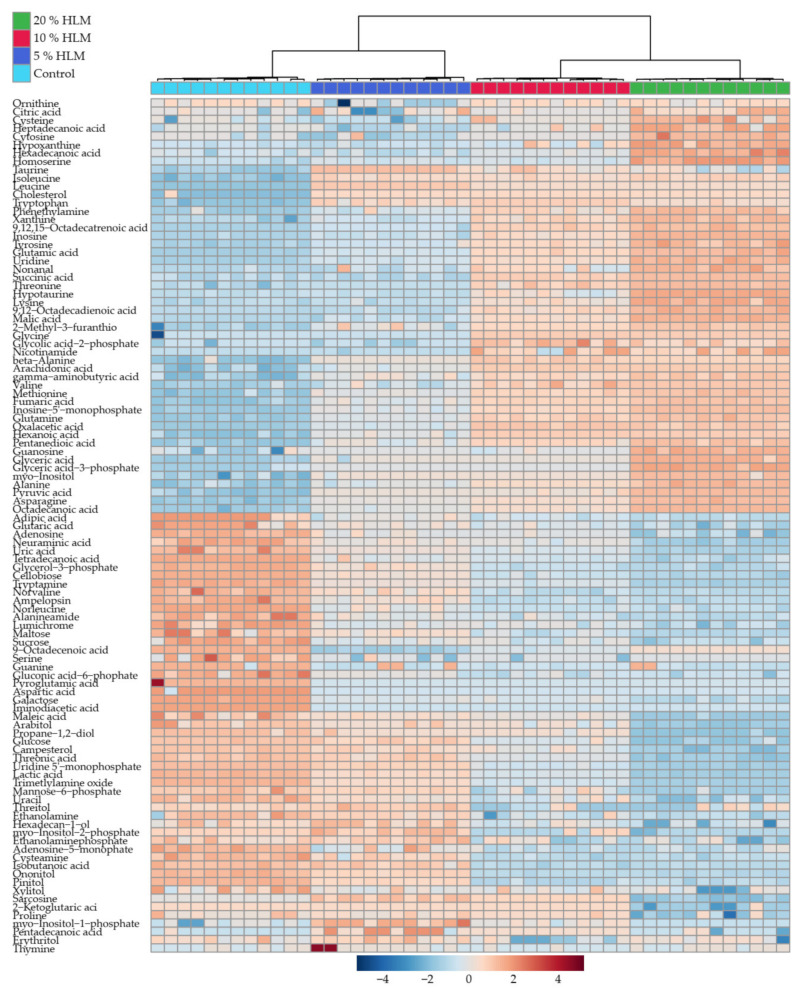
Metabolic profiles of tilapia. The heat map is showing the significant 110 metabolites (*p* < 0.01). This tree diagram has two main branches, each of which includes two major forks. These main branches represent the individual experimental treatments, as indicated by the top color scale. The control, 5%, 10%, and 20% HLM groups are, respectively, distributed in different forks from these two major branches. Red and blue indicate increased and decreased metabolites, respectively, relative to the median abundance level (shown as the bottom color scale).

The 110 identified metabolites were shown in Figure 3 as heatmap, where the basal diet and the 5%, 10%, and 20% HLM feed groups, were distinguished from each other through the clustering dendrogram. This dendrogram had two main branches with two major forks, respectively. These main branches represented the experimental treatments. The 5% HLM groups and the control groups were clustered in one major branch, and distributed in different forks. Similarly, 10% and 20% HLM groups were distributed in different forks from a main branch. As shown in the color scale, the metabolites abundance variation changed with HLM increasing.

### 2.4. Crucial Biomarkers Responsible for H. illucens Larva Meal

**Figure 4:**
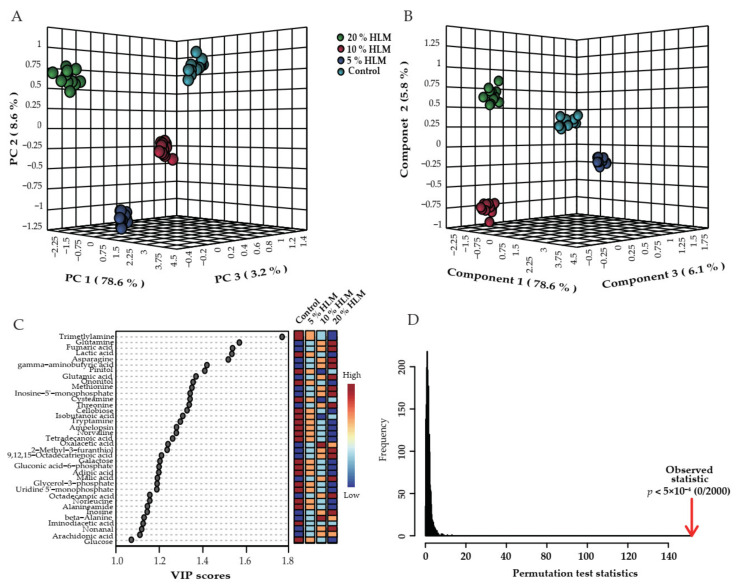
Identification of crucial metabolites. Using unsupervised pattern recognition analysis and supervised pattern recognition analysis to distinguish these groups, PCA (**A**) and PLS-DA (**B**) distinguished the 5%, 10%, and 20% HLM feed and basal diet groups. Each dot represents the technological replicate in these plots. (**C**) VIP plot generates from PLS-DA, and dot represented the candidate biomarkers, whose average abundance was highlighted in color key. There were 37 biomarkers with VIP of component 1 to 3 more than one, respectively. (**D**) In PLS-DA models, 2000 permutation analysis were showing the observed and cross-validated R2 and Q2 coefficients (*p* < 5 × 10^−4^).

To explore the most crucial metabolites differentiating 5%, 10%, and 20% HLM feed from the basal diet groups, an unsupervised pattern recognition analysis principal component analysis (PCA) and a supervised pattern recognition analysis partial least square discriminant analysis (PLS-DA) was conducted to recognize the sample pattern. Results showed that these four groups were distributed in three-dimensional model (accuracy = 1.0, R2 = 0.99498, and Q2 = 0.99117). Principle component 1 to 3 (Figure 4A) and component 1 to 3 (Figure 4B) separated the groups of 5%, 10%, and 20% HLM feed from basal diet groups. A total of PC 1, 2, and 3 in PCA assay explained 90.4% of the global variance which allowed confident interpretation of the variation. What is more, a total of component 1, 2, and 3 used in PLS-DA explained 90.5% of the total variance. Discriminating variables were shown with variable importance for the projection (VIP) plot when cut-off values were set as greater than one in PLS-DA. The biomarkers screened by VIP value of PLS-DA were showed in Figure 4C. There were 37 biomarkers with VIP of component 1 to 3 more than one, such as trimetlylamine oxide, glutamine, fumaric acid, lactic acid, asparagine, gamma-aminobutyric acid, pinitol, glutamic acid, ononitol, methionine, inosine-5’-monophosphate, cysteamine, threonine, cellobiose, isobutanoic acid, tryptamine, ampelopsin, norvaline, tetradecanoic acid, oxalacetic acid, 2-Methyl-3-furanthiol, 9,12,15-octadecatrienoic acid, galactose, gluconic acid-6-phosphate, adipic acid, malic acid, glycerol-3-phosphate, uridine 5’-monophosphate, octadecanoic acid, norleucine, inosine, beta-alanine, iminodiacetic acid, nonanal, arachidonic acid, and glucose. In order to verify the reliability of PLSDA models, 2000 permutation analysis were performed, which were showing the observed and cross-validated R2 and Q2 coefficients (Figure 4D). There was no overfitting of PLS-DA because of *p* < 5 × 10^−4^ in permutation analysis.

### 2.5. Differential Enriched Pathways Responsible for H. illucens Larva Meal

**Figure 5:**
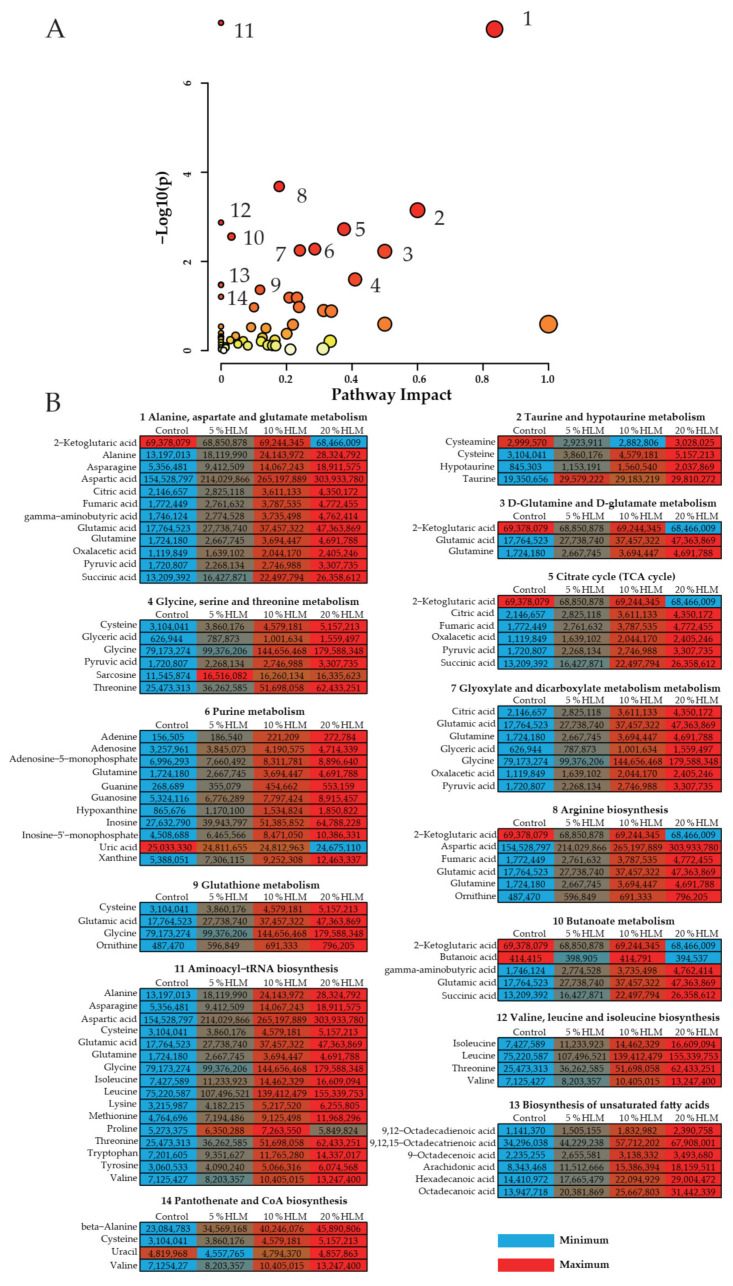
Metabolomic pathway enrichment and analysis. (**A**) There are metabolomic pathways enrichment of varied metabolites in the 5%, 10%, and 20% HLM feed and basal diet control groups (*p* < 0.05). In terms of their impact value from the largest to the smallest, 1 to 14 represented, respectively, following alanine, aspartate and glutamate metabolism; taurine and hypotaurine metabolism; D-glutamine and D-glutamate metabolism; glycine, serine, and threonine metabolism; citrate cycle (TCA cycle); purine metabolism; glyoxylate and dicarboxylate metabolism; arginine biosynthesis; glutathione metabolism; butanoate metabolism; aminoacyl-tRNA biosynthesis; valine, leucine, and isoleucine biosynthesis; biosynthesis of unsaturated fatty acids; pantothenate and CoA biosynthesis. Significant enriched pathways are selected to plot. (**B**) Integrative analysis of metabolites in significantly enriched pathways. The trichromatic diagram was used to distinguish these metabolites’ average abundance. Red and blue represent the maximum and minimum abundances for each row (metabolite), respectively.

This research further investigated which metabolomic pathways were enriched and what significance of the enriched pathways was detected among the 5%, 10%, and 20% HLM feed and basal diet groups. Enriched metabolic pathways are especially important for understanding the metabolomic effects response to the 5%, 10%, and 20% HLM feed and basal diet control. Using MetaboAnalyst 5.0, 14 metabolic pathways were separately enriched these groups (*p* < 0.05) (Figure 5A). According to their impact value, 1 to 14, respectively, represented alanine, aspartate, and glutamate metabolism (*p* = 0.00000006, impact value = 0.84); taurine and hypotaurine metabolism (*p* = 0.0007, impact value = 0.6); D-glutamine and D-glutamate metabolism (*p* = 0.005, impact value = 0.5); glycine, serine, and threonine metabolism (*p* = 0.03, impact value = 0.41); citrate cycle (TCA cycle) (*p* = 0.002, impact value = 0.38); purine metabolism (*p* = 0.005, impact value = 0.29); glyoxylate and dicarboxylate metabolism (*p* = 0.006, impact value = 0.24); arginine biosynthesis (*p* = 0.0002, impact value= 0.18); glutathione metabolism (*p* = 0.04, impact value = 0.12); butanoate metabolism (*p* = 0.003, impact value = 0.032); aminoacyl-tRNA biosynthesis (*p* = 0.00000004, impact value = 0); valine, leucine, and isoleucine biosynthesis (*p* = 0.001, impact value = 0); biosynthesis of unsaturated fatty acids (*p* = 0.03, impact value = 0); pantothenate and CoA biosynthesis (*p* = 0.03, impact value = 0). Of particular interest is the metabolites involved in these 14 pathways (Figure 5B), which are shown the trend of variation by using a diagram. Integrative analysis of metabolites in significantly enriched pathways. The trichromatic diagram was used to distinguish the trend of the average abundance of the metabolites involved in these 14 pathways. Red and blue represent the maximum and minimum abundances for each row (metabolite), respectively.

### 2.6. The Metabolomic Effects Responsible for H. illucens Larva Meal

It was hypothesized that HLM promotes tilapia production. To test this hypothesis, tilapias were fed 5%, 10%, and 20% HLM and the basal diet. These results showed that HLM exerted metabolomic effects in a dose-dependent manner.

**Figure 6:**
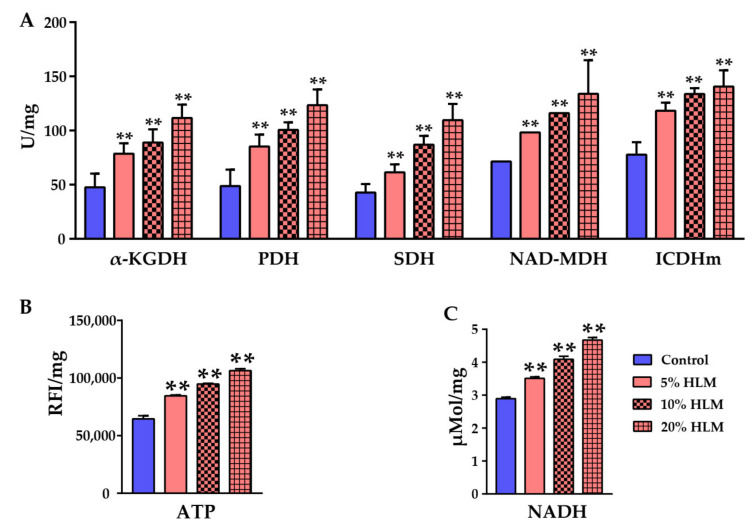
HLM modulates tilapias’ energy metabolism. Compared with the basal diets, there were the activity of ICDHm, NAD-MDH, SDH, PDH, and α-KGDH (**A**), and the ATP (**B**) and NADH content (**C**) of livers in the presence of 5%, 10%, and 20% HLM feeds. Here, ATP was detected by the relative fluorescence intensity (RFI) of probe. ** *p* < 0.01.

To further determine the metabolomic effects of HLM on tilapia production, the enzymatic activities of α-KGDH, PDH, SDH, NAD-MDH, and ICDHm were measured in the tricarboxylic acid (TCA) cycle after 30 days of feeding. As shown in Figure 6, compared with basal diet groups, the enzymatic activity of tilapia were all up-regulated in 5%, 10%, and 20% HLM feed groups. The enzyme activity also increased with an increase in HLM content, and, in detail, the enzymatic activity of α-KGDH (from 47.55 ± 12.77 U/mg of control group, to 78.56 ± 9.66 U/mg and 88.89 ± 12.21 U/mg of 5% and 10% HLM group, respectively, finally up to 111.63 ± 12.2 U/mg of 20% HLM group), PDH (from 48.74 ± 15.26 U/mg of control group, to 85.29 ± 11.02 U/mg and 100.52 ± 7.11 U/mg of 5% and 10% HLM group, respectively, finally up to 123.36 ± 14.49 U/mg of 20% HLM group), SDH (from 42.75 ± 7.89 U/mg of control group, to 61.46 ± 7.25 U/mg and 86.84 ± 8.26 U/mg of 5% and 10% HLM group, respectively, finally up to 109.55 ± 15.03 U/mg of 20% HLM group), NAD-MDH (from 71.45 ± 0.02 U/mg of control group, to 98.25 ± 0.02 U/mg and 116.11 ± 0.04 U/mg of 5% and 10% HLM group, respectively, finally up to 133.98 ± 30.94 U/mg of 20% HLM group), and ICDHm (from 77.68 ± 11.56 U/mg of control group, to 118.21 ± 7.53 U/mg and 133.67 ± 5.48 U/mg of 5% and 10% HLM group, respectively, finally up to 140.67 ± 14.92 U/mg of 20% HLM group) (Figure 6A). Furthermore, the enzymatic activities of TCA cycle were enhanced, thus ATP and NADH levels were determined to confirm the activity of energy metabolism (Figure 6B). Here, ATP was detected by the relative fluorescence intensity (RFI), in detail, the content of ATP (from 64,516.67 ± 2856.05 RFI/mg of control group, to 84,604.17 ± 714.33 RFI/mg and 94,844.17 ± 601.34 RFI/mg of 5% and 10% HLM group, respectively, finally up to 106,488.33 ± 16,295.9 RFI/mg of 20% HLM group) and NADH (from 2.89 ± 0.15 μMol/mg of control group, to 3.51 ± 0.2 μMol/mg and 4.09 ± 0.09 μMol/mg of 5% and 10% HLM group, respectively, finally up to 4.67 ± 0.08 μMol/mg of 20% HLM group). These results suggested that HLM might boost TCA cycle to promote the production performance of tilapia. Therefore, the metabolomic effects of HLM are to enhance the TCA cycle of tilapia fed with 5%, 10%, and 20% HLM feed. Ultimately, the original metabolites of unsaturated fatty acid biosynthesis, amino acid biosynthesis, and glutathione biosynthesis also increased.

**Figure 7:**
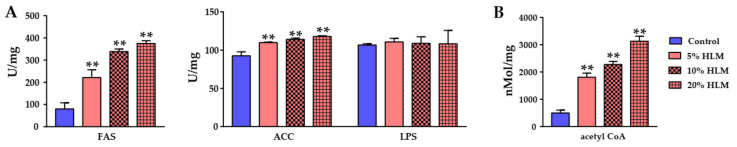
HLM enhances the fatty acids biosynthesis of tilapia. Compared with the basal diets, there were the activity of FAS, ACC, and LPS (**A**), and the acetyl Co-A content (**B**) of livers in the presence of 5%, 10%, and 20% HLM feeds. ** *p* < 0.01.

Furthermore, with an increase in HLM, the abundance of unsaturated fatty acids in liver of tilapia also increased. To investigate the metabolomic effects of HLM on fatty acid biosynthesis of tilapia, the enzymatic activities of FAS and ACC were measured after 30 days of feeding. Compared with basal diet groups, the enzymatic activity of tilapia was all up-regulated in the 5%, 10%, and 20% HLM feed groups. The enzyme activity also increased with increasing HLM content, and, in detail, the enzymatic activity of FAS (from 80.39 ± 27.42 U/mg of control group, to 221.51 ± 34.79 U/mg and 338.51 ± 11.84 U/mg of 5% and 10% HLM group, respectively, finally up to 374.68 ± 12.41 U/mg of 20% HLM group) and ACC (from 92.57 ± 5.16 U/mg of control group, to 109.9 ± 0.55 U/mg and 114.02 ± 1.58 U/mg of 5% and 10% HLM group, respectively, finally up to 117.79 ± 0.97 U/mg of 20% HLM group) (Figure 7A). Conversely, there was no significant change with an increase in HLM content, and in detail, the enzymatic activity of LPS (106.7 ± 1.57 U/mg of control group, and 110.68 ± 4.7 U/mg, 108.91 ± 8.57 U/mg and 108.32 ± 17.49 U/mg of 5%, 10%, and 20% HLM group, respectively). Furthermore, the enzymatic activities of fatty acid biosynthesis were enhanced, thus acetyl Co-A levels were determined to confirm the activity of energy metabolism (Figure 7B), and, in detail, the content of acetyl Co-A (from 497.71 ± 108.33 nMol/mg of control group, to 1806.97 ± 152.65 nMol/mg and 2274.37 ± 115.61 nMol/mg of 5% and 10% HLM group, respectively, finally up to 3129.91 ± 180.92 nMol/mg of 20% HLM group). These results suggest that HLM may boost unsaturated fatty acid biosynthesis to improve the abundance of unsaturated fatty acids. Therefore, the metabolomic effects of HLM are aimed at enhancing unsaturated fatty acid biosynthesis in tilapia fed 5%, 10%, and 20% HLM feed.

**Figure 8:**
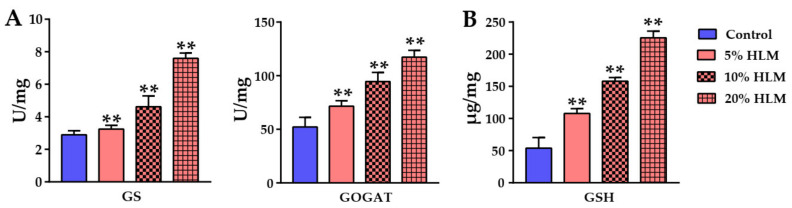
HLM promotes the amino acids biosynthesis of tilapia. Compared with basal diets, there were the activity of GS and GOGAT (**A**), and the GSH content (**B**) of livers fed with 5%, 10%, and 20% HLM feed. ** *p* < 0.01.

With an increase in HLM, the abundance of amino acids in the liver of tilapia also increased. To detect the metabolomic effects of HLM on amino acid biosynthesis of tilapia, the enzymatic activities of GS and GOGAT were measured after 30 days of feeding. Compared with the basal diet groups, the enzymatic activity of tilapia was up-regulated in 5%, 10%, and 20% HLM feed groups. The enzyme activity also increased with increasing HLM content, and in detail, the enzymatic activity of GS (from 2.9 ± 0.24 U/mg of control group, to 3.25 ± 0.24 U/mg and 4.62 ± 0.66 U/mg of 5% and 10% HLM group, respectively, finally up to 7.6 ± 0.32 U/mg of 20% HLM group) and GOGAT (from 52.24 ± 8.92 U/mg of control group, to 71.53 ± 5.1 U/mg and 94.57 ± 8.54 U/mg of 5% and 10% HLM group, respectively, finally up to 117.35 ± 6.33 U/mg of 20% HLM group) (Figure 8A). Moreover, GSH content was also assayed (Figure 8B), which played an important role in reductive metabolic reaction, and, in detail, the content of GSH (from 53.88 ± 16.37 μg/mg of control group, to 107.91 ± 7.34 μg/mg and 158.02 ± 5.58 μg/mg of 5% and 10% HLM group, respectively, finally up to 225.43 ± 10.59 μg/mg of 20% HLM group). These results suggest that HLM may boost amino acid biosynthesis to improve the abundance of amino acids. Therefore, the metabolomic effects of HLM are aimed at enhancing amino acid biosynthesis in tilapia fed 5%, 10%, and 20% HLM feed.

**Figure 9:**
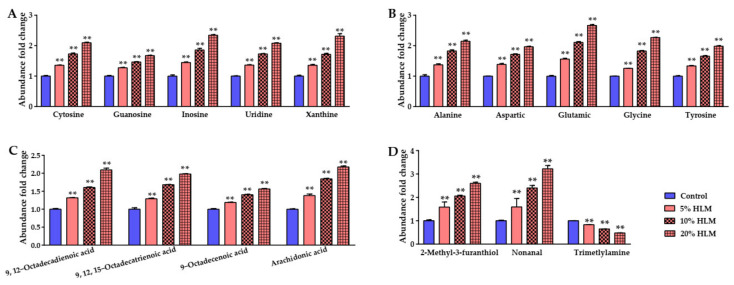
HLM improves the flavor metabolites biosynthesis of tilapia. Here, abundance fold changes of these flavor metabolites were shown with increasing HLM, in detail, flavor nucleotides inosine, guanosine, uridine, cytosine, and xanthine (**A**), flavor amino acids alanine, glutamic acid, aspartic acid, phenylalanine, glycine, and tyrosine (**B**), and flavor fatty acids 9,12-Octadecadienoic acid, 9,12,15-Octadecatrienoic acid, 9-Octadecenoic acid, and arachidonic acid (**C**) totally increased. (**D**) There were the abundance fold changes of flavor metabolites, nonanal, 2-methyl-3-furanthiol, and trimethylamine. ** *p* < 0.01.

The flavor metabolites in the liver of tilapia changed, following an increase in HLM. To highlight its flavor effects, the abundance of flavor metabolites were measured after 30 days of feeding. By comparing the mean GC-MS abundance of the basal diet groups, these flavor metabolites’ GC-MS abundance fold changes were shown in Figure 9 that with increasing HLM, in detail, flavor nucleotides inosine, guanosine, uridine, cytosine, and xanthine (Figure 9A); flavor amino acids alanine, glutamic acid, aspartic acid, phenylalanine, glycine, and tyrosine (Figure 9B); flavor fatty acids 9,12-Octadecadienoic acid, 9,12,15-Octadecatrienoic acid, 9-Octadecenoic acid, and arachidonic acid (Figure 9C) also increased. Figure 9D showed that nonanal and 2-methyl-3-furanthiol with a meaty flavor were also detected, and that their abundance increased with increasing HLM. The details of flavor metabolites were shown in Table S3. In addition, it was notable that, a fishy smell of metabolite trimethylamine oxide (trimethylamine) was detected, and its abundance also decreased with increased HLM. Therefore, the metabolomic effects of HLM promoted the accumulation of beneficial flavor metabolites and reduced the biosynthesis of odor metabolites.

## 3. Discussion

The major challenge for the development of the tilapia farming is the shortage and price increase of feed protein resources (https://www.fao.org/3/i1143e/i1143e.pdf. (accessed on 29 January 2022)). For a long time, raw feed proteins are primarily derived from fish meal of marine fisheries. But this expensive fish meal cannot be used on the cheap tilapia, otherwise it will lead to a loss of tilapia farming (https://www.fao.org/publications/sofia/2020/en/ (accessed on 29 January 2022)). Therefore, there are some studies aimed to explore a beneficial supplements to promote tilapia growth while reducing cost input [5,6,7]. Moreover, these supplements also improve the quality of tilapia meat to meet growing demand of human. Perhaps, insect resources may be an effective response to the crisis [8,9,10,11,12,13,14,15,16,17,18]. It is critical to discover an alternative protein supplement to aquaculture [27]. The increasing human population and intensification of human activity are becoming more pronounced, resulting in a sharp increase in food waste, manure, agricultural waste, etc. *H. illucens* larvae can use these waste products, and breeding *H. illucens* larvae using waste can alleviate the environment of waste pollution [28]. Thus, *H. illucens* larvae can convert waste into high-quality feed supplementation, and Wachira et al. found that HLM supplementation was beneficial to the growth of tilapia [16]. Furthermore, *H. illucens* larvae can provide high-quality protein food source [29]. HLM is not only high in protein content but also in oil content and has high nutritional value [30]. Additionally, *H. illucens* larvae do not carry zoonotic pathogens [8,9]. The soybean meal with removed anti-nutrients still retains such harmful components. However, for cost control, the major commercial tilapia feeds in China use soybean meal instead of fish meal and fish oil. Pervin et al. found that replacing 75% of fish meal with soybean meal did not affect the growth of tilapia [31]. Beneficial effects of replacing fish meal with HLM discovered by Wachira et al. [16]. Therefore, 5%, 10%, and 20% soybean meal in the basal diet were replaced with HLM, respectively, which metabolic effects were revealed through feeding experiments. This study found that the addition of HLM significantly increased the average daily gain of tilapias and the sensory taste of their meat. These results indicated that metabolomic profiling of the HLM feed and control groups was established. The significant changes in metabolome were related to the increase in average daily gain and sensor characteristic. In addition, the key biomarkers and metabolic pathways were identified in different metabolomes, revealing that HLM affects the metabolic level of tilapias, thereby improving production performance.

After feeding tilapia with HLM for a month, there was a significant change in metabolome. The metabolic markers were energy metabolism related metabolites, such as fumaric acid, cellobiose, oxalacetic acid, galactose, gluconic acid-6-phosphate, malic acid, glycerol-3-phosphate, and glucose. The metabolic pathways were significantly changed. Here, TCA cycle and glyoxylate and dicarboxylate metabolism were involved in energy metabolism. Energy metabolism, centered on the TCA cycle, is the key to biosynthetic metabolism, which provides raw materials for important pathways such as amino acid metabolism, fatty acid metabolism, and nucleotide metabolism. With increasing HLM, the activities of ICDHm, NAD-MDH, SDH, PDH, and α-KGDH in the TCA cycle also increased. The enhanced activity of these enzymes improved active anabolism, thereby increasing the average daily gain. As the TCA cycle is activated, the production of ATP and NADH also increases. ATP and NADH are very important substances in metabolic processes and are usually closely related to the production performance of animals. This study found that as the activity of the TCA cycle in the liver of tilapias fed with an increasing HLM, the production of ATP and NADH increased, thereby improving the production performance. Here, Studies have shown that activating the TCA cycle can improve production performance [32], which is consistent with the findings of this study. Interestingly, one study has shown that activating the TCA cycle can improve disease resistance [25,26], which implies that the physique of a tilapia can be enhanced by feeding it with HLM. 

The metabolic markers are fatty acids, such as lactic acid, gamma-aminobutyric acid, isobutanoic acid, tetradecanoic acid, 9,12,15-octadecatrienoic acid, adipic acid, octadecanoic acid, and arachidonic acid. There are significant metabolic pathways involved in fatty acid metabolism, butanoate metabolism; biosynthesis of unsaturated fatty acids; pantothenate and CoA biosynthesis. Because the fat content in HLM is higher than that in soybean meal, replacing soybean meal with HLM improves fatty acid metabolism in tilapia compared with the basal diet. Therefore, one of the major contributions of HLM is to enhance fatty acid metabolism. Fatty acids are an important component of cell membrane, and they are also involved in the formation of meat flavor [33,34]. More importantly, unsaturated fatty acids are also involved in immune regulation [35] and neurophysiological effects [36]. The activities of FAS and ACC in fatty acid biosynthesis were measured and it was found that FAS and ACC activities increased with increasing HLM. Thus, HLM improved active fatty acid metabolism, particularly the biosynthesis of unsaturated fatty acids. In contrast, LPS activity did not change significantly with an increase in HLM quantity fed to fish. It was found that the acetyl CoA content in the liver of tilapias was increased. It can be seen that acetyl CoA does not originate from the fatty acid β-oxidation process but from energy metabolism. Acetyl CoA mainly participates, both, in fatty acid biosynthesis and energy metabolism [36]. Interactively, an increase in acetyl CoA levels were not only increasing the energy metabolic activity, but also promoting the fatty acid biosynthesis.

The metabolic markers, glutamine, asparagine, glutamic acid, methionine, threonine, tryptamine, norvaline, beta-alanine, and norleucine were amino acids. There were significant metabolic pathways involved in amino acid metabolism; alanine, aspartate, and glutamate metabolism; taurine and hypotaurine metabolism; D-glutamine and D-glutamate metabolism; glycine, serine, and threonine metabolism; arginine biosynthesis; glutathione metabolism; aminoacyl-tRNA biosynthesis; valine, leucine, and isoleucine biosynthesis. Amino acid metabolism is the basis of protein biosynthesis. Thus, GS and GOGAT activities in protein biosynthesis were measured and it was found that GS and GOGAT activities also increased with an increase in HLM. This findings are consistent with the performance of the average daily gain. Some studies had conducted experiments in which HLM replaced fish meal, or supplemented in fish diets, and found that it improves the production performance of fish [37,38,39,40,41], which is consistent with the findings of this study. Feeding with HLM promoted amino acid metabolism in tilapias, thereby strengthening protein biosynthesis in tilapias and promoting growth and development. The metabolic pathways were mainly related to amino acid metabolism, and the most notable pathway was glutathione biosynthesis. This pathway was enhanced by the supplements of HLM. Therefore, the GSH content was measured, and it was found that the GSH content in tilapia livers increased. This study further reveals that HLM promotes tilapia growth by strengthening amino acid metabolism. In addition, HLM promotes GSH biosynthesis, which affects these pathways dependent on reduced GSH levels.

The metabolic markers were nucleotide metabolism-related metabolites, such as ononitol, inosine-5’-monophosphate, uridine 5’-monophosphate, and inosine. One of the significantly enriched metabolic pathways involved nucleotide and purine metabolism. Nucleotide metabolism is the basis for the biosynthesis of genetic materials. This study involves enriched purine metabolism, and it was found that as the amount of HLM was increased, the abundance of purine metabolites also increased, thus indicating that the tilapia’s average daily gain increased significantly along with the increase in genetic material biosynthesis. This study further revealed that HLM feed promotes tilapia growth by enhancing nucleotide metabolism in tilapias. Moreover, studies have shown that purine metabolism-related metabolites can improve disease resistance [42,43], indicating that feeding HLM may enhance the production performance and premunition of tilapia.

Factory farming of tilapias has greatly increased tilapia meat production. Thus, people’s pursuit of fish flavors has become increasingly prominent [44]. Nucleotides, amino acids, and unsaturated fatty acids have a flavor effect which can increase meat flavor, tasty smell, and sweet taste [45]. With an increase in HLM, flavor nucleotides inosine, guanosine, uridine, cytosine, and xanthine; flavor amino acids alanine, glutamic acid, aspartic acid, phenylalanine, glycine, and tyrosine; flavor fatty acids 9,12-Octadecadienoic acid, 9,12,15-Octadecatrienoic acid, 9-Octadecenoic acid, and arachidonic acid also increased. The metabolites, nonanal [46] and 2-methyl-3-furanthiol [47], with a meaty flavor, were detected using the GC-MS platform, and their abundance also increased with increasing HLM. In addition, it was notable that a fishy smell of metabolite trimethylamine oxide [48] was detected, and its abundance also decreased with increased HLM. Generally, the liver is the most powerful digestive organ and involved in principle metabolic activities of the animal. Metabolites from the liver are transported to the muscle via the blood, where the metabolites are further metabolized. Therefore, the metabolomic level of liver determined the flavor and quality of muscle [49]. These results portended that HLM further affected muscle flavor through hepatic metabolism in the digestive system. 

In summary, this study uncovered the important metabolomic effects that could promote the production performance and the meat flavor of Nile tilapia. After feeding with HLM diets, tilapias’ metabolome was boosting via these enhanced pathways of energy metabolism, amino acid and protein biosynthesis, fatty acid biosynthesis, and purine metabolism. Most importantly, HLM stimulated the enzymatic activity, and increased the content ATP, NADH, acetyl CoA, and GSH in the liver of tilapia. Furthermore, these beneficial metabolomic responses reduced the synthesis of off-flavor trimethylamine and increased umami metabolites in the liver. Thus, HLM might be a crucial factor in regulating metabolism in tilapias, and a good feed protein supplement.

## 4. Materials and Methods

### 4.1. Ethics Statement

According to the Guide for the Care and Use of Laboratory Animals of the National Institutes of Health and its standard protocols, this work was carried out and abided by its recommendations. All experiments in this study were approved by the Institutional Animal Care and Use Committee of Zhongkai University of Agriculture and Engineering (ZK20200803).

### 4.2. Chemical Reagents

The enhanced BCA protein assay kit, ATP and NADH content determination kit were purchased from Beyotime Institute of Biotechnology (catalog: P0012, S0026 and S0175, Shanghai, China). Fatty acid synthase (FAS), acetyl CoA carboxylase (ACC), lipase (LPS), mitochondrial isocitrate dehydrogenase (ICDHm), NAD-malate dehydrogenase (NAD-MDH), succinate dehydrogenase (SDH), pyruvate dehydrogenase (PDH), α-ketoglutarate dehydrogenase (α-KGDH), glutamine synthetase (GS), glutamate synthase (GOGAT), acetyl Co-A, and GSH assay kits were purchased from Beijing Solarbio Science & Technology Company Limited (Beijing, China), which catalog were BC0555, BC0415, BC2345, BC2165, BC1045, BC0955, BC0385, BC0715, BC0350, BC0075, BC0985, and BC1175, respectively. Chemical reagents were obtained by Shanghai Aladdin Bio-Chem Technology Company Limited (Shanghai, China). Deionized water (18.9 MΩ) was obtained by filtration of distilled water using a Milli-Q system (Millipore, MA, USA).

### 4.3. Feed Preparation

According to the commercial formula feed for tilapia (catalog: 6653, Charoen Pokphand Group, Zhanjiang, China) and nutritional standards for Nile tilapia formula feed (SCT1025-1998, China), HLM and other feed ingredients are purchased from the feed market of Guangzhou. These ingredients were pulverized and pressed into particles as the basal diet excluded HLM, which components are shown in Table S1. Here, mineral and vitamin premix were purchased by commercial production of (Chelota^®^, Chelota Biotechnology Group Company Limited, Deyang, China. License number: Sichuan feed premix (2015) 05005) and (Yulong^®^, Guangzhou Southern Biotechnology Company Limited, Guangzhou, China. License number: Guangdong feeding (2020) 01131. Executive standard: Q/NFSW 19-2021). Then, referring to this commercial feed formula and nutritional standard, its soybean meal was replaced with 5%, 10%, and 20% HLM as the experimental group, whilst it was ensured that the total energy of these feeds was not significantly different from that of the control group, in detail, 17.431 KJ/Kg of basal diet, 17.432, 17.432, and 17.433 KJ/Kg of 5%, 10%, and 20% HLM feed, respectively. A dry extruding method was used in feed process. In detail, using a pulverizer (360, Yugong Technology Development Company Limited, Xingtai, China), the raw materials (Table S1) were, respectively, pulverized into powder and then sieved to remove impurities that could not be pulverized. After mixing together excluded premix, the major components were pulverized again and sieved to remove impurities. These mixtures were evenly mixed together after adding the mineral and vitamin premix. An automatic extruder (DGP60-C, Yugong Technology Development Company Limited, Xingtai, China) was employed to extrude these mixtures into granules. Then these granules were dried at 65 °C for 1 h (Electric heating constant temperature blast drying oven, DHG-9053A, Zhongxin Medical Instrument Company Limited, Jiaxing, China) until the water content was lower than 10%. Finally, these diets were containing non-significant crude fiber (14.51%), water (9.61%), and crude ash (14.56%). There were the crude protein content of these feeds, in detail, 31.25% of basal diet, 31.97%, 32.68%, and 33.41% of 5%, 10%, and 20% HLM feed, respectively. What is more, there were the crude fat content of these feeds, in detail, 3.72% of basal diet, 4.07%, 4.49%, and 4.87% of 5%, 10%, and 20% HLM feed, respectively. The total phosphorus content were, respectively, 0.86%, 0.89%, and 0.94% in 5%, 10%, and 20% HLM feed, compared with 0.82% in basal diet. Moreover, methionine, cystine, and lysine relative content were, respectively, 0.53%, 0.41%, and 1.69% in 5% HLM feed, 0.56%, 0.45%, and 1.72% in 10% HLM feed, 0.63%, 0.49%, and 1.78% in 20% HLM feed, compared with 0.48%, 0.39%, and 1.61% in basal diet.

### 4.4. Fish and Rearing Conditions

The body weight of Nile tilapia is 60.00 ± 5.35 g (mean ± SEM) obtained from a tilapia breeding corporation (Guangzhou, China) and maintained in 120 L (full volume 150 L) open-circuit water tanks with aeration. These fish were free of specific pathogen through microbiological detection. Tilapia was cultured as described previously [25,26,50]. The water temperature at 25 °C, the dissolved oxygen content at 8.75 mg/L, and the water pH 7.0 were maintained during rearing. The water circulation and filtration system was always on to keep the water clean. Based on NRC recommendations, these fish were fed with 3% of their body weight per meal. These tilapias were fed three times a day and the residual feed should be removed one hour after feeding. If tilapias have a lot of feces and little or no residual feed, these fish were re-fed once. These tilapias were kept at this condition for 30 days before collection of samples. There were three tanks in each experimental group, and 12 tilapias were reared in each tank. In this study, a total of 144 tilapias were used. After 30 days of feeding, these 114 tilapias were weighted and calculated average daily gain. The average daily gain was calculated as the daily average of the total weight of each group after feeding minus the total weight before feeding. Among these 114 tilapias, two fish were randomly selected from each tank, so that a total of six fish per group were selected for metabolomic sample preparation and detection. In the same way, six fish per group were randomly selected from three tanks for enzymatic testing. When this work had been conducted, the rest fish were released.

### 4.5. Sample Preparing for Gas Chromatography–Mass Spectrometry (GC-MS) Detection

According to the guidelines of National Institutes of Health, tilapias were euthanized in a mixture of ice slush and water (5 parts ice slush into 1 part water, 0–4 °C) for at fifteen min following cessation of gill movement, and then left in the ice water mixture for twenty min after cessation of all life activity to ensure death by hypoxia. These tilapias were rinsed with sterile pure water and then wiped thoroughly with sterilized gauze. Six livers were removed ascetically, where 50 mg of livers were cut and immersed immediately in 1000 μL cold methanol (−20 °C). Then, these samples were sonicated for 5 min at 260 W-power setting at Ultrasonic Processor (JY92-IIDN, Ningbo Scientz biotechnology Company Limited, Ningbo, China), and centrifuged at 14,167× *g* in 4 °C for ten min. The supernatants were collected and ten µL ribitol (100 μg per mL, catalog: A103030, Shanghai Aladdin Bio-Chem Technology Company Limited, Shanghai, China) was added into each sample tube as an internal quantitative standard. Then these supernatants were concentrated in a centrifugal vacuum concentrator (Labconco Company Limited, Kansas City, MO, USA). These dried extracts were derivatized for GC-MS detection. In detail, these dried extracts were dissolved in 0.1 mL solution, 20 mg per mL of methoxamine hydrochloride in pyridine (catalog: M300386 and P111513, Shanghai Aladdin Bio-Chem Technology Company Limited, Shanghai, China), and incubated for two hours at 37 °C in an incubator shaker. Then, 0.1 mL N,O-Bis (trimethylsilyl)trifluoroacetamide (TMSTFA, catalog: B118473, Shanghai Aladdin Bio-Chem Technology Company Limited, Shanghai, China) with 1% trimethylchlorosilane was added into the solutions and incubated for half an hour at 37 °C. There were six biological replicates for each experimental treatment.

### 4.6. Gas Chromatography–Mass Spectrometry Detection

After derivatization, 1 μL was injected per sample into an HP-5MS capillary column (Agilent Technologies Company Limited, Santa Clara, CA, USA. 30 m × 250 μm × i.d. 0.25 μm. Part number: 19091S-433UI) using splitless injection and detection was carried out by GC-MS detector (7890A equipped with 5975C VL, Agilent Technologies Company Limited, Santa Clara, CA, USA). The initial temperature of GC oven was held at 85 °C for five min followed by an increase to 280 °C at a rate of fifteen °C per min holding for five min and then increased to 310 °C at a rate of twenty °C per min. The solvent delay time was set as five min before signal acquisition. High purity helium (purity more than 99.999%) was used as a carrier gas and flow rate was kept constantly at 1 mL per min. Using full scan (SCAN) mode, MS was operated in a range of 50–600 m/z.

### 4.7. Mass Spectra Processing for Gas Chromatography–Mass Spectrometry

The deconvolution and calibration of these acquired mass spectra were performed with AMDIS (version: OpenLAB CDS ChemiStation C.01.01, Agilent Technologies Company Limited, Santa Clara, CA, USA). In order to avoid false positives, these peaks with a signal-to-noise ratio (S/N) higher than 30% were retained for subsequent analysis [51]. Additionally, compared with the blank reagent samples, these artifact peaks were excluded. Metabolites were identified by retrieving their characteristic ion fragments in the NIST 2011 (National Institute of Standards and Technology, USA. Version: MS search 2.0) library and GMD 2011 (Golm Metabolome Database, Germany. Version: GMD_20111121_MDN35_ALK) referring to the following criteria: match value greater than 750, reverse match value greater than 800, and a probability more than 60% [52]. Moreover, the metabolites’ mass spectra detected by this study were compared with the characteristic ion fragments in the HMDB database (https://hmdb.ca/ (accessed on 29 January 2022)). Kovats retention index (RI) was measured with 34 n-alkanes reference (catalog: CDAA-M-690038-HD-1mL. Anpel Laboratory Technologies Incorporated, Shanghai, China), whilst all metabolites was derivatized by oximation of methoxamine hydrochloride and silanization of N,O-Bis(trimethylsilyl)trifluoroacetamide. The relative peak area value of the internal standard ribitol (m/z 152.0685) was taken to calculate these metabolites abundance. After removal of ribitol and any known artificial peaks, and integration of the same compounds, the metabolites’ abundance with reliable signal were detected in each sample. These zeroes or missing values were assessed by singular value decomposition (SVD) method to output the missing values [53]. Variables were excluded for threshold 50%, and missing values of each group were replaced with their respective medians value. Then, interquartile rage (IQR) filtering was improved these data [54]. Here, the combined normalization processing consisted of the following options [55]: logarithmic transformation and quantile normalization row-wise procedures combined with pareto scaling (mean-centered and divided by the square root of standard deviation of each variable). The data array created an Office Excel file can be used for the subsequent multivariate statistical analysis.

### 4.8. Detection of the Enzyme Activity and Measurement of the Contents of Glutathione, Reduced Nicotinamide-Adenine Dinucleotid, and Adenosine Triphosphate

Six liver samples were detected. Then, enzyme activity assay and acetyl CoA contents were measured as the manual of assay kits of Beijing Solarbio Science & Technology Company Limited (http://www.solarbio.net/. (accessed on 29 January 2022)). Additionally, glutathione (GSH), adenosine triphosphate (ATP) and reduced nicotinamide-adenine dinucleotid (NADH) content was measured as the manual of assay kits of Beyotime Institute of Biotechnology (https://www.beyotime.com/goods.do?method=getallcplist&flag=1&lang=english. (accessed on 29 January 2022)). Tilapias’ livers were rapidly sampled after euthanasia with ice slush water. In brief, one gram liver sample were rinsed with 10 mL 1 × PBS (50 mM, pH 7.4; Invitrogen, Thermo Fisher Scientific China Company Limited, Shanghai, China), resuspended in lysate buffer, and homogenized by a homogenizer (TGrinder catalog: OSE-Y40. Tiangen Biotech Company Limited, Beijing, China) for five min at 340 W power. After centrifugation at 14,167× *g* in 4 °C for ten min, the supernatants were transferred to a new tube. The crude enzyme amount was calculated based on the total protein content, and then the total proteins concentration in the supernatants was determined by BCA kit (P009, Beyotime Biotechnology Company Limited, Shanghai, China). The total proteins of 200 µg were applied for the activity assay of FAS, ACC, LPS, ICDHm, NAD-MDH, SDH, PDH, α-KGDH, GS, GOGAT, and the contents determination of acetyl Co-A, GSH, NADH, and ATP. The enzymatic assay and content determination was conducted by the Kits instructions. In detail, it is detected to mix an equal volume of the crude enzyme and reaction buffer to a final volume of 200 µL in 96-well plates. These plates were further incubated at 25 °C for fifteen min, and the absorbance was read on a spectrophotometer (Synergy HTX, BioTek Instruments Company Limited, Winooski, VT, USA). The enzymatic activity was evaluated by plotting against the standard curve or control. All determinations were conducted in duplicates. The values expressed are the mean of measurements. *t*-test was used as the significance analysis.

### 4.9. Bioinformatic Analyses

Using Office Excel, data array transformations and manipulations were performed. The significance in the metabolites abundance among these groups were compared by the ANOVA (α = 0.01) with the SPSS 23.0 (IBM Corporation, Armonk, NY, USA). Here, a multivariate statistical analysis of the normalized data array was further performed using the MetaboAnalyst 5.0 (www.metaboanalyst.ca (accessed on 29 January 2022), Ste. Anne de Bellevue, QC, Canada) [56,57]. Using the distance matrix calculated with the Euclidean method, a hierarchical cluster analysis (HCA) was first performed. Here, the unsupervised principal component analysis (PCA) and supervised partial least squares-discriminant analysis (PLS-DA) was then conducted to investigate the relationships among these groups. Based on the variable importance for the projection (VIP) value of major PLS-DA components, these metabolites with VIP value greater than one, were filtered and shown in a color scale scatter plot. 

For the metabolomic pathway enrichment analysis, these pathways involving the metabolites that showed variances between these HLM groups were identified using the MetaboAnalyst 5.0. In details, the metabolomic pathway analysis algorithms were over representation analysis (hypergeometric test) and pathway topology analysis (relative-betweenness centrality). Using a pathway library (KEGG version Oct 2019), the model animal was selected as *Danio rerio* (KEGG No.7955). Other conditions as default, and then metabolomic pathways enrichment were performed. Please specify a reference metabolome: use all compounds in the selected pathways. Demonstrating significant metabolic pathways with a bubble chart, the raw *p* value and an impact value of each pathway were evaluated using a hypergeometric test, and these significant metabolic pathways with *p* < 0.05 were retained.

The nonparametric Kruskal–Wallis one-way analysis combined with Dunn multiple comparison post hoc test was employed for the data processing of enzymatic activity. In statistical analysis, *p* < 0.05 and *p* < 0.01 was considered significantly. What is more, it is used to draw the histogram by Prism v7.0 (GraphPad, La Jolla, CA, USA). 

### 4.10. Sensory Evaluation

All recruited volunteers were informed of the methods and aims of this sensory evaluation, then signed an informed consent. According to Pu et al. [58], twenty volunteers (ten men and ten women aged between 18 and 21, healthy and non-smoking without taste/odor disorders) were trained by the ranking test for three weeks. The taste solutions including sweetness standard glucose (50.00 g/L), sourness standard citric acid (5.00 g/L), saltiness standard sodium chloride (15.00 g/L), umami standard sodium glutamic acid (20.00 g/L), and bitterness standard sodium quinine (6.00 g/L) were, respectively, diluted in 1/2, 1/4, 1/6, 1/8, and 1/16. Especially, the fishy smell test is trained by sniffing. Each of the taste solutions with five differential concentrations was presented to tasting panel, and these volunteers were asked to rank the orders according to taste perception intensity. After a single-blind test, only these volunteers who answered correctly can participate in the further trial. Here, 1000 mL pure water was added into 100 g fresh tilapia meat samples, and boiled for 10 min. Then, these 50 mL aquatic extraction samples were filtered and cooled for testing. Referring to the sensory evaluation standard (GB/T16291.1-2012, Sensory analysis—Methodology-magnitude estimation method, China), these samples were put into a bottle and randomly numbered with three groups. In the umami, bitterness, sweetness, sourness, and saltiness sensory analysis, volunteers were asked to take a sip of the sample and to keep it in the mouth for 10 s, then spit it out. The intensity of each attribute (sweetness, sourness, bitterness, saltiness and umami) was scored by the volunteers. At the interval between samples, volunteers should be asked to wash their mouth with 50 mL drinkable water for three times to avoid fatigue and carryover effect. This sensory evaluation test was performed on different groups at one hour-interval. 

## Data Availability

The data presented in this study are available in the article and Supplementary Materials. The GC-MS raw data required to reproduce these findings cannot be shared at this time as the raw data also forms part of an ongoing study. Additionally, the GC-MS raw data are available on request from the corresponding author.

## Supplementary Materials

The following supporting information can be downloaded at: https://www.mdpi.com/article/10.3390/metabo12040286/s1, Table S1: Feed formula of basal diet used in this study, Table S2: The initial and final weight of average daily gain measurement, Table S3: Flavor composition of tilapia.
