# Supplementary material for: Metabolomic Effects of the Dietary Inclusion of Hermetia illucens Larva Meal in Tilapia
Source: Metabolites. 2022 Mar 24;12(4):286. doi: 10.3390/metabo12040286 (PMC9032204; doi:10.3390/metabo12040286)
Supplement: Supplementary file 1 [file metabolites-12-00286-s001.zip › metabolites-1597676-supplementary.pdf]

metabolites

Table S1. Feed formula of basal diet used in this study

Component	Content (%)
Soybean meal (with hulls)	35
Wheat middling	10
Barley malt sprouts	17.5
Brewer's grain	10
Alfalfa meal	10
Wheat bran	10
Palm oil	2
Monocalcium phosphate	2
Mineral premix	1
Bentonite	1
Gelatinized starch (corn)	0.28
Vitamin premix	1
NaCl	0.2
Ethoxyquin	0.02

Vitamin premix is purchased by a commercial production (Yulong®, Guangzhou Southern Biotechnology Company Limited, Guangzhou, China. License number: Guangdong feeding (2020) 01131. Executive standard: Q/NFSW 19-2021). Mineral premix is purchased from feed manufacturer (Chelota®, Chelota Biotechnology Group Company Limited, Deyang, China. License number: Sichuan feed premix (2015) 05005). HLM and other feed ingredients are purchased from the feed market of Guangzhou.

Table S2. The initial and final weight of average daily gain measurement.

No.	Control		5% HLM		10% HLM		20% HLM	
	**Initial weight (g)**	**Final weight (g)**	**Initial weight (g)**	**Final weight (g)**	**Initial weight (g)**	**Final weight (g)**	**Initial weight (g)**	**Final weight (g)**
1	711	1152.05	728.24	1437.66	740.04	1849.61	714.09	2463.48
2	712.94	1177.2	717.3	1389.33	729.85	1905.28	735.53	2540.89
3	700.99	1145.47	728.96	1472.9	740.04	1926.79	678.74	2556.75

No. means that the number of replicates for each treatment group. There were 12 tilapias per group.

Table S3. Flavor composition of tilapia.

Flavor compound	RT (min)	RI	HMDBID	Abundance (mean ± SEM)			
				**Control**	**5% HLM**	**10% HLM**	**20% HLM**
Cytosine	17.64	1525	0000630	2.03E+06 ± 4.55E+04	2.76E+06 ± 1.82E+04	3.52E+06 ± 5.77E+04	4.26E+06 ± 3.36E+04
Guanosine	40.57	2792	0000133	5.32E+06 ± 1.23E+05	6.78E+06 ± 6.78E+04	7.80E+06 ± 5.57E+04	8.92E+06 ± 6.65E+04
Inosine	37.54	2624	0000195	2.76E+07 ± 1.17E+06	3.99E+07 ± 5.85E+05	5.14E+07 ± 1.40E+06	6.48E+07 ± 4.97E+05
Uridine	41.27	2442	0000296	2.74E+06 ± 3.11E+04	3.73E+06 ± 4.05E+04	4.74E+06 ± 3.31E+04	5.71E+06 ± 4.22E+04
Xanthine	28.25	2121	0000292	5.39E+06 ± 1.61E+05	7.31E+06 ± 1.56E+05	9.25E+06 ± 1.54E+05	1.25E+07 ± 4.50E+05
Alanine	7.05	1153	0000161	1.32E+07 ± 6.39E+05	1.81E+07 ± 4.77E+05	2.41E+07 ± 4.81E+05	2.83E+07 ± 4.63E+05

Aspartic acid	17.61	1348	0000191	1.55E+08 ± 1.17E+06	2.14E+08 ± 4.98E+06	2.65E+08 ± 3.13E+06	3.04E+08 ± 2.16E+06
Glutamic acid	17.67	1508	0000148	1.78E+07 ± 4.59E+05	2.77E+07 ± 4.86E+05	3.75E+07 ± 5.34E+05	4.74E+07 ± 5.10E+05
Glycine	7.41	1071	0000123	7.92E+07 ± 3.79E+05	9.94E+07 ± 3.16E+05	1.45E+08 ± 1.41E+06	1.80E+08 ± 4.50E+05
Tyrosine	25.33	1962	0000158	3.06E+06 ± 4.66E+04	4.09E+06 ± 5.48E+04	5.07E+06 ± 6.19E+04	6.07E+06 ± 5.05E+04
9,12-Octadecadienoic acid	31.32	2214	0247706	1.14E+06 ± 2.53E+04	1.51E+06 ± 6.78E+03	1.83E+06 ± 1.87E+04	2.39E+06 ± 5.53E+04
9,12,15-Octadecatrienoic acid	30.91	2212	0001388	3.43E+07 ± 1.39E+06	4.42E+07 ± 5.85E+05	5.77E+07 ± 2.33E+05	6.79E+07 ± 2.73E+05
9-Octadecenoic acid	31.54	2218	0062703	2.24E+06 ± 4.61E+04	2.66E+06 ± 2.11E+04	3.14E+06 ± 2.59E+04	3.49E+06 ± 3.32E+04
Arachidonic acid	38.73	2369	0001043	8.34E+06 ± 8.87E+04	1.15E+07 ± 3.64E+05	1.54E+07 ± 1.35E+05	1.82E+07 ± 2.16E+05
2-Methyl-3-furanthiol	7.23	1077	0036611	1.91E+06 ± 7.39E+04	3.02E+06 ± 4.14E+05	3.92E+06 ± 7.36E+04	4.96E+06 ± 9.18E+04
Nonanal	8.52	1323	0059835	2.53E+05 ± 6.28E+03	4.02E+05 ± 9.20E+04	6.07E+05 ± 2.66E+04	8.14E+05 ± 3.55E+04
Trimetlylamine	8.17	748	0032539	5.69E+06 ± 3.07E+04	4.73E+06 ± 4.28E+04	3.69E+06 ± 3.24E+04	2.73E+06 ± 4.22E+04

RI: Kovats retention index (RI). RT is the retention time in minutes. Abundance values of GC-MS detection represent relative content in these samples, with a unit of 1, where the abundance of each group is expressed as mean ± SEM.
